# Retraction: The effects of residual platelets in plasma on plasminogen activator inhibitor-1 and plasminogen activator inhibitor-1-related assays

**DOI:** 10.1371/journal.pone.0202381

**Published:** 2018-08-09

**Authors:** The PLOS ONE Editors

Following publication of this article [1], concerns were raised regarding the reporting of the methodology for the first sub-study that used samples collected from participants in the SABPA study [2].

The article states that: “Samples were randomly divided into two groups. One half of the study population samples were centrifuged at 352 *g* and the other half at 1500 *g* for 15 minutes at 20°C to yield plasma containing a varying number of platelets.”

The corresponding author has informed the journal that the SABPA study samples were collected over two years, with samples collected from Black African participants in 2008 and samples collected from White African participants in 2009. Data collection from the SABPA samples was completed before the subsample of 151 individuals was selected for inclusion in the current study. Randomization and centrifugation at different speeds was not carried out as part of the study design. Differences in PAI-1 and βTG levels were observed between the samples collected in 2008 and those collected in 2009, and a discrepancy in the written laboratory protocol raises the possibility that different centrifuge speeds had been used in different years; however, SABPA investigators state that despite a discrepancy in the written protocol which stated “1500 rpm (2000 *g*)”, the same centrifuge settings were used in both years, 1500 rpm using a rotor diameter of 12.9cm, which equates to 324 *g* (Hettich 320, Andreas Hettich GmbH & Co. KG). No determination is made by the *PLOS ONE* Editors regarding the actual centrifugation conditions used.

Following evaluation of the issues identified, the *PLOS ONE* Editors understand that the data reported from the follow-up study in the second part of the article support the conclusions drawn; however, we have determined that the study design and methodology reported for the SABPA sub-study was misrepresented, and information about the SABPA study samples that may affect the interpretation of the results was omitted. In light of the above issues, the *PLOS ONE* Editors retract this article.

MP agreed with the retraction. SAB, DTL, DCR did not respond.
